# Biomechanical Analysis of the Fixation System for T-Shaped Acetabular Fracture

**DOI:** 10.1155/2015/370631

**Published:** 2015-10-01

**Authors:** Yanping Fan, Jianyin Lei, Feng Zhu, Zhiqiang Li, Weiyi Chen, Ximing Liu

**Affiliations:** ^1^Institute of Applied Mechanics and Biomedical Engineering, Taiyuan University of Technology, 79 Yingze West Street, Taiyuan 030024, China; ^2^Bioengineering Center, Wayne State University, Detroit, MI 48201, USA; ^3^Department of Orthopedics, Wuhan General Hospital of Guangzhou Command, 627 Wuluo Road, Wuhan 430070, China

## Abstract

This study aims to evaluate the biomechanical mechanism of fixation systems in the most frequent T-shaped acetabular fracture using finite element method. The treatment of acetabular fractures was based on extensive clinical experience. Three commonly accepted rigid fixation methods (double column reconstruction plates (P × 2), anterior column plate combined with posterior column screws (P + PS), and anterior column plate combined with quadrilateral area screws (P + QS)) were chosen for evaluation. On the basis of the finite element model, the biomechanics of these fixation systems were assessed through effective stiffness levels, stress distributions, force transfers, and displacements along the fracture lines. All three fixation systems can be used to obtain effective functional outcomes. The third fixation system (P + QS) was the optimal method for T-shaped acetabular fracture. This fixation system may reduce many of the risks and limitations associated with other fixation systems.

## 1. Introduction

Acetabular fractures are frequently associated with high-impact trauma, particularly trauma incurred from road traffic accidents. Pelvic fractures frequently involve injury to organs contained within the bony pelvis because of the impact of forces involved. Furthermore, trauma to extrapelvic organs is common and pelvic fractures are often associated with severe hemorrhage because of the extensive blood supply to the region. The mortality rate of patients with pelvic fractures is between 10% and 16%. This type of injury often causes enormous damage to society and families. Therefore, a precise diagnosis and a well-executed treatment plan are important in achieving functional and durable results [1].

The displaced T-shaped fracture is complicated to manage and is frequently encountered (commonly from motor vehicle accidents, cycling accidents, or fall from significant heights). Open reduction and internal fixation with interfragmentary screws and reconstruction plates are the treatment of choice in the displaced posterior wall and posterior column fractures of the acetabulum [2]. Early anatomical reduction with rigid fixation is the immediate goal of surgical treatment. The specific problems associated with internal fixation include the intra-articular penetration of screws or loss of fixation [3–5], particularly because malnutrition can result in the accelerated degradation of the articular cartilage [6]. Anatomical reduction and stable fixation are essential for effective and long-term clinical results [3, 5]. However, one-fifth of patients with simple or additional complex fractures exhibit poor results [7].

Studies on the biomechanical mechanism, practicality, and effectiveness of internal fixation systems are rare because of the complexity of the pelvic fracture and its fixation systems. The finite element method (FEM) has the intrinsic advantages of restricting individual differences without the need for equipment and environmental variations. In biomechanical analysis by using the FE model, any combination of models and any change of external loads are possible. The analysis of the FE model can provide the local reaction mechanism, investigate the stress-strain status in any surface and inner regions of the model, and avoid the restrictions and deviations caused by the use of cadaver tests [8]. Therefore, this analysis serves as a valuable supplement to clinical observations and autopsy studies.

This study aims to create an FE model and evaluate the biomechanical mechanism of three methods of fixation systems for T-shaped fracture, one of the most common fractures with complicated procedures. The biomechanics of these fixation systems were assessed on the basis of effective stiffness levels, stress distributions, force transfers, and displacements along the fracture lines.

## 2. Materials and Methods

### 2.1. FEM of the Pelvis

**Figure 1:**
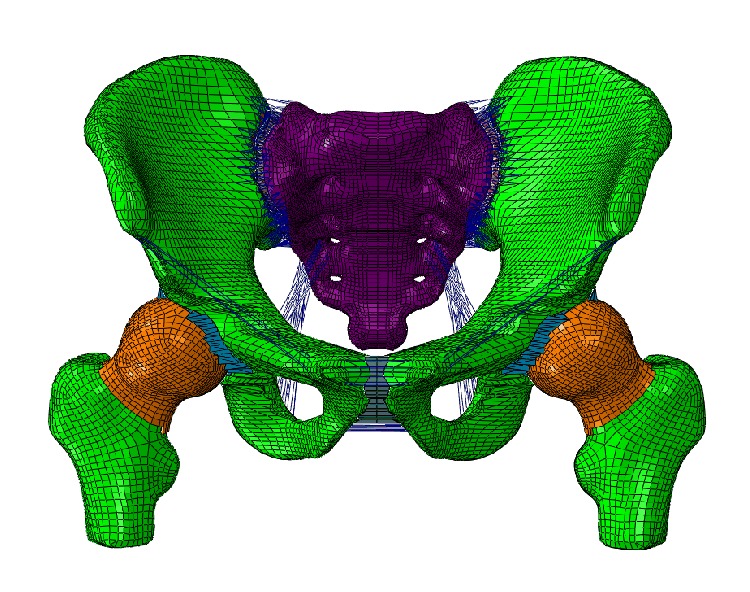
FE model of the pelvis.

The hospital ethics committee licensed this study. A 3D FE model of the pelvis was created on the basis of the computed tomography (CT) scan images with a slice width of 0.5 mm of a Chinese male (40 years old, 175 cm tall, and 65 kg weight). The point cloud was converted to the surface of the pelvic. Mesh division was conducted on each part of the tissue by a combined manual and automatic division method. Eight nodes with nonlinear solid hexahedron elements (C3D8), with an average thickness of 1.5 mm, were offset from the cancellous bone to represent cortical bone. The soft tissues (i.e., endplates, cartilage, and pubic symphysis) between pelvic bone were meshed into hexahedron elements. The bones, cartilages, and endplates were all represented by hexahedral mesh. To make the simulation realistic, ligaments including sacroiliac ligament ring, sacrospinous, sacrotuberous, inguinal, superior pubic, and arcuate pubic represented by truss elements were added to the FE model. The main pelvic ligaments were modeled as truss elements with a length of 2 mm. In addition, the material properties of the model were assumed to be homogeneous and isotropic. The total numbers of elements and nodes were 102506 and 147458, respectively. Furthermore, mesh sensitivity studies revealed that further refinement did not significantly improve calculation accuracy. The FE model of the pelvis is shown in Figure 1. The material properties of the pelvis are shown in Table 1.

### 2.2. Fragment Model

**Figure 2:**
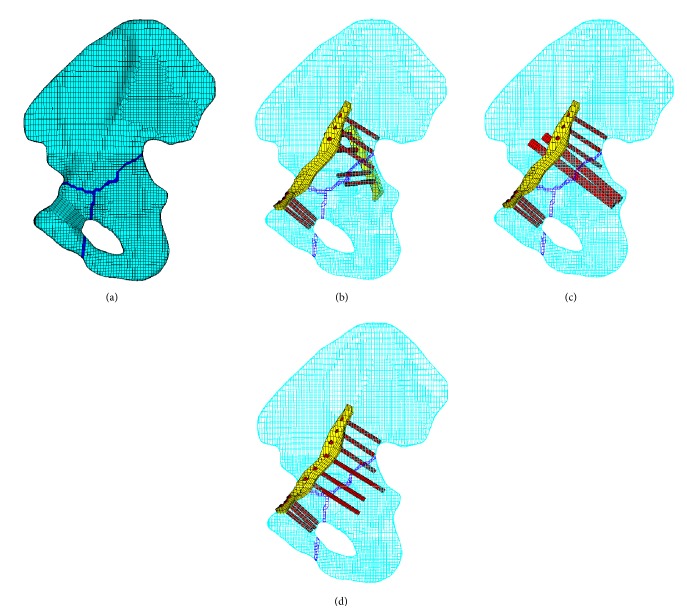
Finite model of three fixation systems; (a) fracture model without fixation systems; (b) double column reconstruction plates (P × 2); (c) an anterior column plate combined with posterior column screws (P + PS); (d) an anterior column plate combined with quadrilateral area screws (P + QS).

A T-shaped fracture combines a transverse component and vertical component that separates the lower ischiopubic segment into the anterior and posterior columns. This fracture can be categorized as a combined fracture. In this paper, a converging line was developed to represent the fracture line. The converging line originated from the anterior inferior iliac spine or groove on the upper brim of the acetabulum and runs along the center of the acetabulum to separate to two branches: one to the anterior side of the acetabulum and the other reaching the inferior side of the acetabulum. To simulate the instability of the pelvis, the inferior ramus was also ruptured as shown in Figure 2(a).

T-shaped fractures separate the pelvis into three parts. The upper part of the pelvis cannot be kept stable because of the lower parts of the pelvic that serve little or even no effect on support of the body weight. The pelvis suffers lesser stiffness compared with the intact bone. In this case, the properties (i.e., density and elastic modulus) of the mesh along the converging line were weakened to one-tenth of the normal mesh [9, 10].

### 2.3. Surgical Techniques

The type and nature of the acetabular fracture substantially influence the approach used [11]. The goal of the operation was to achieve an anatomical reduction of the innominate bone and the articular surface of the acetabulum. A prospective clinical evaluation of such cases was conducted wherein the main surgical strategy was open reduction and internal fixation with reconstruction plates and screws. The outcome measures of the operation included the magnitude of initial displacement, and the postoperative values indicated the amount of reduction achieved [11]. The fixation system could reposition the fracture component into the original position to achieve a good therapeutic effect. A fracture fixation device made of nitinol (NiTi) alloy is more beneficial to fracture healing; thus, the fixation systems are made from this material [2].

#### 2.3.1. Double Column Reconstruction Plates (P × 2)

This fixation system was the most traditional and widely used method compared with the other two systems, particularly in impacted and osteochondral fragments. This fixation system consisted of two reconstruction plates and its set screws [9]. For anterior column fixation, a pelvic reconstruction plate was used that extended from the pubic symphysis across the rim of the pelvis spanning the anterior column defect, ranging 10 cm to the medial wall of the ilium. Posterior column fixation was applied from the outer surface of the ilium, next to the acetabular rim into the body of the ilium (see Figure 2(b)). The plates had to be bent into an appropriate shape before application to the iliac crest and were fixed by several set screws. The interface between the plates and bone was modeled as face-to-face contact with a frictional coefficient of 0.1 to simulate the slide between the joint surfaces [11]. Care was taken to avoid the intersection of the screws when inserted to secure the plates. The reconstruction plates were simplified as smoothed plates, excluding the screw holes. The threads of the screws were simplified, and the screws were simulated with 3.5 mm diameter rod-like structures.

#### 2.3.2. An Anterior Column Plate Combined with Posterior Column Screws (P + PS)

Longer operation time, infection, greater blood loss, abductor weakness, and heterotopic ossification should be avoided during the surgery. Thus, a minimally invasive approach with one reconstruction plate was applied for the complex fracture. The anterior column plate (P) and its set screws were the same as the former (P × 2) system. Furthermore, two lag screws were incorporated from the outer surface of the quadrilateral area superior to the arcuate line and into the ischial spine under screw fixation. The two lag screws should be completely immersed in the iliac bone and not through the acetabulum cartilage to avoid extra damage to the patient (see Figure 2(c)). The lag screws were simplified as 6.5 mm diameter set screws.

#### 2.3.3. An Anterior Column Plate Combined with Quadrilateral Area Screws (P + QS)

The position attaching the reconstruction plate in this fixation system was almost the same as the two former (P × 2 and P + PS) systems. The quadrilateral area screws (QS) were inserted from the outer surface along the arcuate line into the ischial spine. The QS were fixed by the reconstruction plates and the quadrilateral area cortical bone where they were inserted (see Figure 2(d)). The QS were also secured by the extrusion force generated by the interference fit between the screws and holes. This fixation may create a more stable effort than the former system because the elasticity modulus and density of the cortical bone were larger than the trabecular bone.

The purpose of this study was to evaluate the validation of the fixation systems of the T-shaped fracture in double-limb stance. This stance was similar to the existing models, as previously described by Sawaguchi et al. [12]. The model was placed in a specific neutral position defined with iliac wings level (coplanar in the horizontal plane). In the sagittal plane, the proximal femoral shaft was vertical. In the sagittal plane, the proximal femoral shaft was vertical. A value of 600 N represents that the upper body weight distributed uniformly on the upper surface of the sacral bone. The degrees of freedom of the end of the femur were restrained to represent the double-limb stance.

## 3. Results

### 3.1. Validation of the Pelvic Model

**Figure 3:**
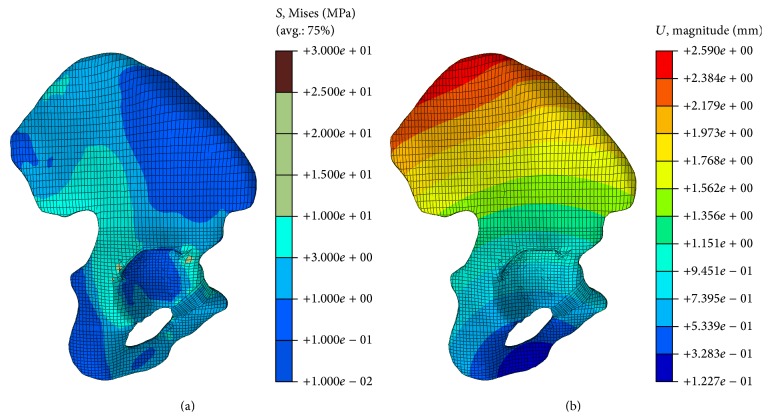
Stress and displacement distribution in the cortical bone of iliac bone; (a) stress distribution in the cortical bone of iliac bone; (b) displacement distribution in the cortical bone of iliac bone.

The pelvic model experiences a vertical force loaded on the upper surface of the sacral bone. The von Mises stress and displacement distribution in the iliac bone are shown in Figure 3. The present study corroborated the findings of Anderson et al. [13] and Phillips et al. [14]. The maximum displacement value was 2.59 mm in our paper, compared to 2.2 mm in the Shi' studies [8]. The regions of stress concentration were observed at the superior rim of the acetabulum and on the ilium superior to the acetabulum. The ranges of value of the von Mises stress and displacement are similar across the studies, and the difference is attributed to the load or boundary conditions or the properties of the bone, such as the ignorance of the sacrum, femur, and soft tissues (i.e., cartilage and endplate). These findings show that the FE model developed in this study was validated in terms of stress and displacement.

### 3.2. Validation of the Fixation Systems

The rigidity of five different configurations (nonfractured model, fracture model, and three fixation systems models), which were obtained from simulated results, was compared with each other (Table 2). The fracture model without fixation systems achieves a higher maximum displacement than the other conditions. Therefore, the effective stiffness of the fracture model is unable to bear a large force. The max von Mises stress is a signal of the stress concentration; that is, a larger max stress corresponds to a more severe stress concentration. The fracture model without fixation systems is more unstable than other situations. Thus, this model suffers the most severe stress distribution. The third fixation system (P + QS) has the most stable condition, whereas the first fixation system (P × 2) achieves an even stress distribution owing perhaps to the screws inserted. The QS inserted in the cortical bone plays a greater role in supporting the fractured bone than the other screws immersed in the low-density trabecular bone. The first fixation system (P × 2) constitutes a maximum number of plates and screws for the fixation system to smoothen the stress effectively.

**Figure 4:**
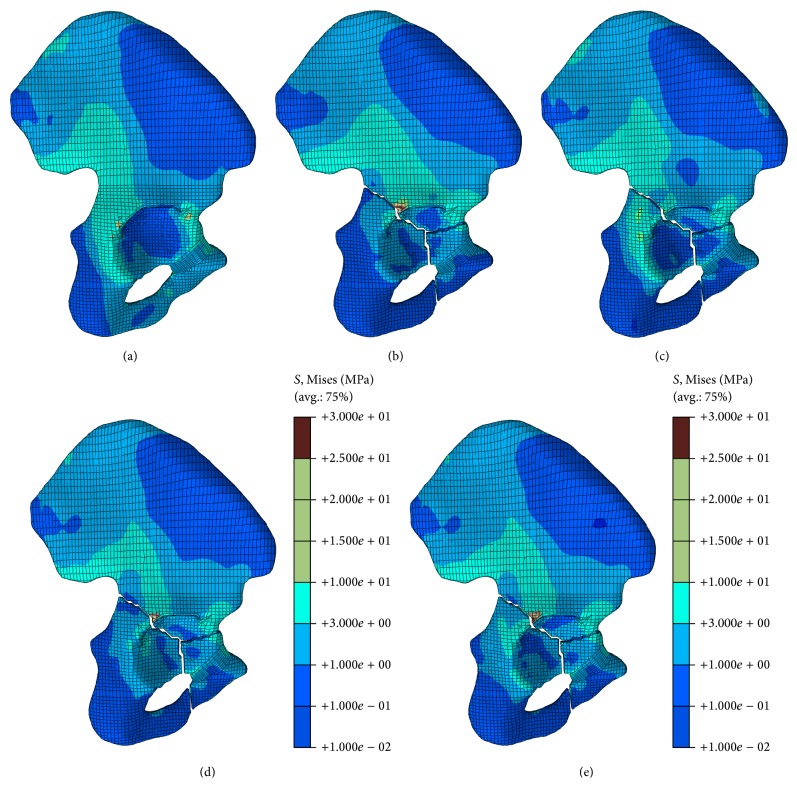
Stress distribution in the iliac bone under different conditions; (a) stress distribution in the iliac bone in the nonfractured model; (b) stress distribution in the iliac bone in the fracture model; (c) stress distribution in the iliac bone in the first fixation system (P × 2); (d) stress distribution in the iliac bone in the second fixation system (P + PS); (e) stress distribution in the iliac bone in the third fixation system (P + QS).

The stress distributions in the iliac bone are shown in Figure 4. In the nonfractured model, the highest stress occurs at the sacroiliac joint. The tuberculum pubicum also suffers considerable stress fields, which are the stress distribution type when the fracture occurred. The stress in the fracture corner is considerably high, with the highest stress occurring at the same place where the fracture lines run across the limbus of the acetabulum. The highest stress was decided by the fixation system type. The stress distributed in most parts of the iliac bone was lower than that in the nonfractured model. Therefore, the stress level in the fracture model is lower than the value in the nonfractured model. The occurrence of the fracture changed the load transfer path. A major difference is that the load transfer from the outer surface of the ilium is expected of the fracture model. All these fixation systems serve good functions for the stability of the T-shaped fracture.

**Figure 5:**
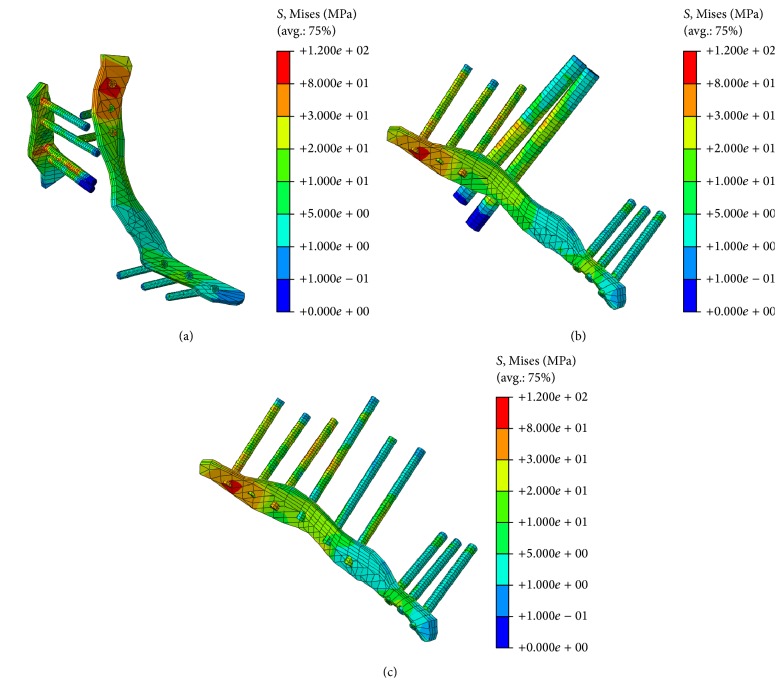
Stress distribution in different fixation systems; (a) stress distribution in the first fixation system (P × 2); (b) stress distribution in the second fixation system (P + PS); (c) stress distribution in the third fixation system (P + QS).

The stress distribution patterns in each fixation system under the standing stance are shown in Figure 5. The highest stress region is noted at the middle of the screws attached to the cancellous bone fracture line, and no stress was observed at the end of the screws. This phenomenon can be seen in almost all screws, in addition to lag screws. Higher stress in the reconstruction plates was noted in the regions where the screws were tied, particularly for the set screws. The role of the reconstruction plate can be shown in all systems: the plates all suffered higher stress than the iliac bone. Therefore, the reconstruction plate was placed to keep the artificial surface of the intact bone. The lag screws suffer the same stress distribution in the latter two fixation systems. The stress for the lag screws in the third fixation system (P + QS) is larger than the values of other screws, including the PS and set screws. Therefore, QS served the function of keeping the pelvic fracture in a stable situation.

**Figure 6:**
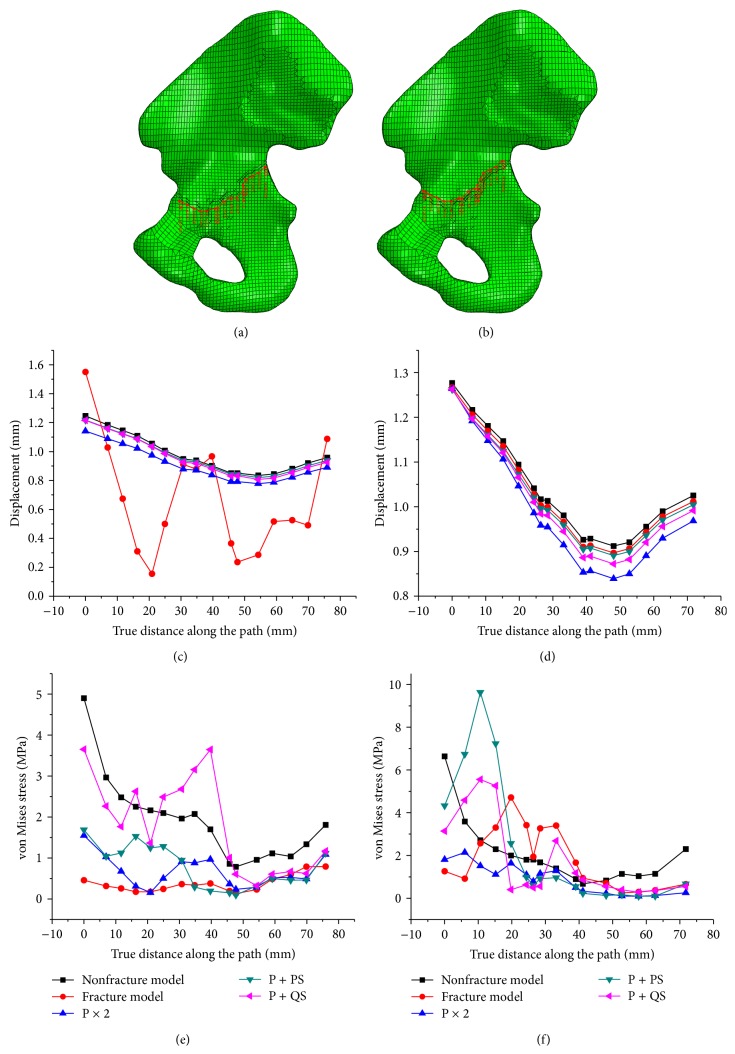
Magnitude of displacement and stress distribution along two paths; (a) the path below the fracture line; (b) the path above the fracture line; (c) magnitude of displacement along the first path; (d) magnitude of displacement along the second path; (e) stress the along first path; (f) stress along the second path.

The two paths were generated along the fracture line to access the validation of the fixation systems. One path is below the fracture line and the other above the fracture line. The displacement along two paths was shown in Figures 6(c) and 6(d). It shows that all conditions contain some common displacement patterns, except for the fracture model. This may be caused by the change in the load transfer path because of the appearance of the fracture. The stress distributions along the path are shown in Figures 6(e) and 6(f). The fixation system influenced the stress level and distribution. A conclusion can be made that all fixation systems were able to keep the fracture into an intact bone. The difference of the displacement along the first line (downward the fracture line) was lower than that along the second line (upward fracture line). This result may be due to the fact that the degrees of femur freedom were restrained; thus, the lower separated part of the pelvis attached to the femur suffers a relatively small deformation when compared with the upper separated part of the pelvis.

## 4. Discussion

This study aims to simulate the mechanical behavior of the T-shaped fracture and assess the biomechanical mechanism of the fixation systems recommended for fracture stabilization. The biomechanical mechanism was evaluated by the effective stiffness, stress distribution, and force transformation of the three models.

A number of approaches have been used to predict stress patterns in biomechanical applications, for example, experimental techniques such as strain gauging and photoelastic analysis and numerical procedures such as FEM, to obtain comprehensive information on the pelvis biomechanical mechanism. The versatile features of FEM analysis are its potential for evaluating stresses/strains throughout the pelvis for all materials concerned and for parametric analysis. Material properties and loading and boundary conditions are easily varied to investigate their influences. Thus, FEM has been selected as a tool to investigate the effects of different fixation systems on complex pelvic T-shaped fractures.

The treatment of pelvic fractures was based on extensive clinical experience and theories that formed the procedures and guidelines for the treatment of fractures. Considering the geometry and structure of the pelvis, one of the most popular systems in surgery is anterior column fixation, which involves the inner surface of the ilium and the superior surface of the superior pubic ramus. The single reconstruction plate is applicable to almost all pelvic fractures, whereas the anterior reconstruction plate alone cannot achieve an acceptable clinical or radiological outcome [1, 3, 15–19]. Therefore, some other fixation systems, such as another plate or PS, were added to reduce the risk of failure of the system.

Erkmen et al. conducted an FE analysis to estimate the complex stress fields in the pelvic bone, fixation screws, and plates and to evaluate the function of the fixation screws and plates [20]. Pierannunzii et al. used double plates or iliac and iliopubic plates to treat the fracture in both columns. They claimed that these two systems were suitable approaches for fractures. They also reported that the fundamental elements of pathoanatomy and radiology would influence treatment planning and surgical intervention [18]. Sawaguchi et al. conducted cadaveric experiments to compare the differences of two fixation systems; they found that the reconstruction plate, which is readily contoured to the intricate periacetabular bony structure, showed no significant difference in rigidity compared with the other apparently more rigid plates [12]. Boccaccio et al. recommended the use of buttress plates with screws for the posterior wall fracture, particularly in younger patients [9]. All of these studies showed that reconstruction plates with interfragmentary or lag screws were the choice for the treatment of displaced pelvic fractures.

The bone in the quadrilateral area is very thin and presents an almost all cortical bone feature. The bone is also proximal to the joint. This particular bone has a high incidence of all types of pelvic fracture. Traditional clinical operations address these fractures without involving the bone in the quadrilateral area to prevent introducing unnecessary risks, whereas extensive clinical experience suggests that the fixation system involved in this area can provide great function outcomes [21]. Maintaining the stability of the pelvic bone is important and depends on the blocking effect of the acetabular bone [22]. The blocking effect of the cortical bone was larger than the value of the cancellous bone. Thus, the screws inserted in the quadrilateral area render the bone more stable than the procedure wherein the screws were immersed in the bone.

The stress and displacement distributions changed when the fracture occurred. The stress level in most parts of the iliac bone in the fracture models were lower than that in the nonfractured model. The stress levels increased significantly in all of the positioned plates attached or screws inserted. Therefore, fixation systems can increase the stiffness of the entire pelvis. The function of the fixations can be explained from the stress distribution pattern: higher stress in the fixation system component corresponds to the greater role played by this component. The maximum stress was observed in the reconstruction plate or the QS; thus, the plate and QS played a dominant role in keeping the stability of the fracture model. The stress level in the fixed screws was lower than in the PS and QS perhaps because the lag screws (PS and QS) penetrated through the fracture line, which fit closely to the irregular surfaces to overcome the resistance generated by shear and torsion. Therefore, the reconstruction plates combined with the lag screws can produce excellent functional outcomes for complex pelvic fractures [21].

Compared with the other two fixation systems (P × 2 and P + PS), the third fixation system (P + QS) can increase the total stiffness and decrease the maximum displacement. This fixation system can transfer more body weight from the upper separated part to the downward separated part through the plate than the other two systems. These findings may be explained by the fact that the elastic modulus of the fixation system was considerably greater than that on the cortical bone (110 GPa versus 17 GPa). Therefore, this fixation (P + QS) is the optimization method for T-shaped fracture in terms of the total pelvic stiffness, stress distribution, and screws role.

The results in this paper were based on the FE model. A few points should be noted. First, the geometry and structure of the pelvis was complex. The pelvis included many sharp corners and small clearance spaces, which cannot be simulated in the FE model. Previous studies have shown that the total pelvic mechanism was insensitive to these detailed features [13]. Furthermore, truss element type with elastic modulus rather than nonlinear characteristics was used to simulate the pelvic ligaments. The elastic approximation is accurate enough for a comparative study of the stability of the pelvis [23]. Second, the T-shaped fracture line changed stochastically, thus making it impossible to find a universal fixation system for this fracture [21]. Moreover, the validation of the surgery depended on many factors such as operative time, estimated blood loss, fluid replacement, blood product replacement, occurrence of intraoperative complications, and recovering after operation. The practicality of the fixation systems, including the intra-articular penetration of screws, the buckling of the reconstruction plates, and loss of fixation systems, deserves our major attention. Therefore, further basic research on the assessment of pelvic injury should be conducted in the future to reach a precise conclusion.

## 5. Conclusion

This study aims to assess the biomechanical mechanism of the fixation systems for T-shaped fracture. Three fixation systems selected in this study are powerful on increasing the approximate biomechanical stability. The third fixation system (P + QS) is the optimal method for T-shaped fracture in terms of total pelvic stiffness, stress distribution, and screw function. Furthermore, this fixation system entails a short operation time, minor cuts, and low possibility of infection during the surgery. Further clinical studies are needed to validate the observations of the current FE study.

## Figures and Tables

**Table 1:** The material properties of the pelvic tissues [8].

	Material	*E* (MPa)	*υ*	Thickness (mm)
Bones	Cortical bone	17000	0.3	1.50
	Cancellous bone	150	0.2	

Soft tissues	End plate (sacrum)	24	0.4	0.23
	Cartilage (sacrum)	54	0.4	3.00
	Cartilage (ilium)	54	0.4	1.00
	End plate (ilium)	24	0.4	0.36
	Pubic symphysis	5	0.495	

Ligamenta	Sacroiliac ligament ring	350	0.3	
	Sacrospinous	29	0.3	
	Sacrotuberous	33	0.3	
	Inguinal	2.6	0.3	
	Superior pubic	19	0.3	
	Arcuate pubic	20	0.3	

Fixations	screws	110000	0.3	
	Plates	110000	0.3	

**Table 2:** Effective stiffness levels of the fixation systems.

	Displacement (mm)	Total stiffness (N/mm)	Max von Mises stress (MPa)
Nonfractured model	2.590	231.66	27.9
Fracture model	2.702	222.06	64.0
P × 2	2.616	229.36	28.7
P + PS	2.645	226.84	35.2
P + QS	2.607	230.15	37.8

## Abbreviations

P × 2: Double column reconstruction plates
P + PS: Anterior column plate combined with posterior column screws
P + QS: Anterior column plate combined with quadrilateral area screws.
